# Pre-extensively Drug-Resistant Congenital Tuberculosis in an Extremely Premature Baby

**DOI:** 10.1093/cid/ciad540

**Published:** 2023-09-08

**Authors:** Alison Boast, Jeu Ann How, Charis Lau, Arun Sett, Damien Gilby, Andrew Burke, Brett McWhinney, Connor Wright, Adrian Tramontana, Maria Globan, Justin Denholm, Stephen M Graham, Joshua Osowicki

**Affiliations:** Department of Paediatrics, University of Melbourne, Parkville, Victoria, Australia; Infectious Diseases Unit, Department of General Medicine, Royal Children's Hospital Melbourne, Parkville, Victoria, Australia; Antimicrobials Research Group, Murdoch Children's Research Institute, Parkville, Victoria, Australia; Newborn Services, Joan Kirner Women's and Children's Hospital, St Albans, Victoria, Australia; Newborn Services, Joan Kirner Women's and Children's Hospital, St Albans, Victoria, Australia; Pharmacy Department, Joan Kirner Women's and Children's Hospital, St Albans, Victoria, Australia; Department of Obstetrics and Gynaecology, University of Melbourne, Parkville, Victoria, Australia; Newborn Research Centre, The Royal Women's Hospital, Parkville, Victoria, Australia; Centre of Research Excellence in Newborn Medicine, Murdoch Children's Research Institute, Parkville, Victoria, Australia; Newborn Services, Joan Kirner Women's and Children's Hospital, St Albans, Victoria, Australia; Department of Thoracic Medicine, The Prince Charles Hospital, Chermside, Queensland, Australia; Centre for Clinical Research, Faculty of Medicine, The University of Queensland, Brisbane, Australia; Department of Chemical Pathology, Pathology Queensland, Queensland Health, Herston, Australia; Department of Infectious Diseases, Western Health, St Albans, Victoria, Australia; Department of Infectious Diseases, Western Health, St Albans, Victoria, Australia; Mycobacterium Reference Laboratory, Victorian Infectious Diseases Reference Laboratory, at the Peter Doherty Institute for Infection and Immunity, Parkville, Victoria, Australia; Victorian Tuberculosis Program, Melbourne Health, Parkville, Victoria, Australia; Department of Infectious Diseases, University of Melbourne at the Peter Doherty Institute for Infection and Immunity, Melbourne, Victoria, Australia; Department of Paediatrics, University of Melbourne, Parkville, Victoria, Australia; Department of Paediatrics, University of Melbourne, Parkville, Victoria, Australia; Infectious Diseases Unit, Department of General Medicine, Royal Children's Hospital Melbourne, Parkville, Victoria, Australia; Tropical Diseases Research Group, Murdoch Children's Research Institute, Parkville, Victoria, Australia

**Keywords:** congenital tuberculosis, multidrug resistant, premature, neonate, therapeutic drug monitoring

## Abstract

We describe a case of congenital tuberculosis in an extremely premature baby, with rapid molecular detection of a pre-extensively drug-resistant (XDR) pattern of drug resistance. The baby was treated successfully with a regimen including bedaquline and delamanid, drugs not previously described in the treatment of congenital tuberculosis (TB).

Congenital tuberculosis (TB) is well described but uncommonly reported [1]. Recognized risks include undiagnosed maternal disseminated and genitourinary TB during pregnancy [2]. Early detection and treatment of maternal TB can protect the fetus and newborn from congenital TB and preterm delivery, but TB-related symptoms are often masked by pregnancy [1, 3]. There are few reports of congenital drug resistant (DR) TB.

Most pre-extensively drug-resistant (XDR) TB can now be treated with all-oral regimens. Pre-XDR TB is defined as rifampicin resistant or multidrug resistant TB (resistant to both rifampicin and isoniazid) plus fluoroquinolone resistance. XDR-TB is defined by further resistance to bedaquiline or linezolid [4]. However, the lack of data for newer drugs such as bedaquiline, delamanid, and pretomanid has excluded young children from recently revised recommendations. This has made it difficult to treat DR TB, particularly fluoroquinolone-resistant TB, without injectable drugs associated with permanent hearing loss [3]. In March 2022, the World Health Organization (WHO) published recommendations for the use of the bedaquiline and delamanid in children of all ages, with dosage guidance for infants weighing 3 kg or greater [5]. For preterm or low birth weight neonates (under 2.5 kg) there are no data to inform the use of newer drugs. Drugs administered to premature neonates may have altered pharmacokinetics compared to older children, and the impact of this on DR TB medications is uncertain [6].

## CASE PRESENTATION

A 943-gram male newborn was delivered vaginally at 26 weeks’ gestation following spontaneous preterm labour. His mother migrated to Australia from India 3 years earlier. She had been well during the pregnancy except for episodes of vaginal bleeding and a 2-week history of cough prior to delivery. Initial management of the infant included high-flow nasal prong respiratory support and brief empiric antibiotic treatment for episodes of presumed (culture negative) sepsis. Routine cranial ultrasonography showed a grade 4 intraventricular hemorrhage.

Nine days post-partum, the baby's mother was readmitted to hospital with fever, abdominal pain, cough, and headache. Histopathological examination of retained products of conception identified chronic active necrotizing granulomatous endometritis, consistent with TB. A chest computer tomography scan to investigate the persistent cough showed miliary changes and her condition evolved to reveal TB meningitis. As the maternal diagnosis of disseminated TB became apparent, so did a previously undisclosed history of 6 months of treatment for pulmonary TB in India 10 years earlier.

**Figure 1.**
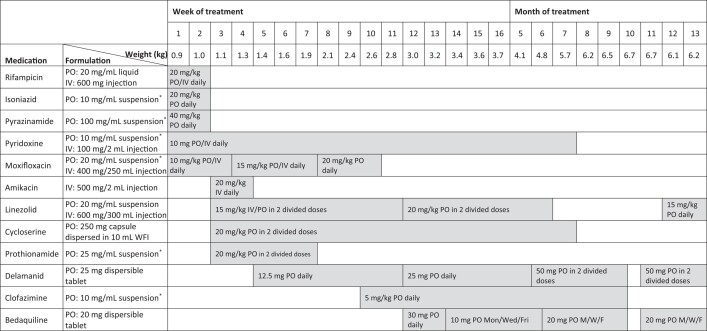
Summary of Treatment. Abbreviations: IV, intravenous; M/W/F, Monday/Wednesday/Friday; PO, oral (including enteral administration via orogastric or nasogastric tube); WFI, water for injection. *Extemporaneously compounded by the Pharmacy Department using tablets that were commercially available in Australia or sourced from overseas via the Special Access Scheme.

On day 15 of life the baby had investigations for congenital TB, and empiric anti-TB therapy was commenced. Cerebrospinal fluid studies had a normal protein without pleocytosis. Empiric anti-TB therapy was commenced with isoniazid, rifampin, pyrazinamide, and moxifloxacin (Figure 1). Moxifloxacin was preferred over ethambutol given its relatively higher bactericidal activity, greater penetration to sites of interest including CNS, and inability to monitor ocular side-effects. Shortly after commencing empiric therapy, the patient was intubated for hypoxia and apnoeic episodes and required high frequency oscillatory ventilation, muscle relaxation, vasopressors, and low then moderate dose corticosteroids [7]. Serial chest X-rays showed evolving cystic changes.

On day 26 of life, Xpert MTB/RIF Ultra assay (Cepheid Sunnyvale, CA, United States) on maternal paraffin-embedded endometrial tissue detected *Mycobacterium tuberculosis*-complex DNA and a mutation in the *rpoB* gene. A subsequent Xpert MTB/XDR assay (Cepheid Sunnyvale, CA, United States) detected additional mutations associated with resistance to isoniazid and fluoroquinolones, and the infant's treatment was adjusted (Figure 1). Rifampin, isoniazid, and pyrazinamide were ceased, and linezolid, cycloserine, prothionamide, and amikacin were commenced (Figure 1). Delamanid replaced amikacin following a successful compassionate use application. Moxifloxacin was continued while awaiting phenotypic DST to determine low- or high-level resistance and while newer bactericidal drugs were being procured. Culture and molecular assays also detected *M. tuberculosis* in infant blood, urine, gastric, and respiratory samples (Supplementary Table 1).

Eventually, phenotypic drug susceptibility testing (DST) showed resistance to isoniazid, rifampin, fluoroquinolones, ethionamide (discordant with the Xpert XDR assay), ethambutol, and pyrazinamide (Supplementary Table 2). Whole genome sequencing (WGS) did not identify a recognized mutation for pyrazinamide and ethionamide resistance (Supplementary Table 3). Moxifloxacin and prothionamide were replaced by clofazimine and bedaquiline. Similarly, the mother's regimen included bedaquiline, pretomind, and linezolid (ie, BPaL). Therapeutic drug monitoring (TDM) with dose adjustment was prospectively undertaken for the infant, with measured plasma concentrations that were generally consistent with therapeutic concentrations described in older cohorts (Supplementary Appendix).

The infant's corticosteroid therapy was continued until extubation on day 32 of life. He was discharged at 7 months of age (4 months corrected gestational age) on low-flow nasal prong oxygen for chronic lung disease of prematurity with a nasogastric tube for enteral nutrition. At discharge he weighed 6.5 kg (10th centile on Fenton premature growth chart). After 6 months of treatment, linezolid and cycloserine were stopped prior to discharge, to arrive at a planned continuation phase regimen of bedaquiline, delamanid, and clofazimine, all administered via nasogastric tube. The infant's mother was treated and discharged with a similar regimen of clofazimine, cycloserine, bedaquiline, and linezolid. Discharge planning included arrangements for regular allied health and medical reviews and visits to the home by the Victorian Tuberculosis Program.

The family were counseled about the side effects of medications and the infant had regular clinical examination (including formal dilated retinal examination) and blood monitoring. Adverse effects included transient neutropenia, which resolved without linezolid dose-adjustment, and unilateral sensorineural hearing loss. No abnormalities (including QT interval) were detected on regular electrocardiograms, which began twice weekly once high-frequency oscillatory ventilation was stopped.

Anti-tuberculous treatment was briefly interrupted at 10 months of age, due to vomiting temporally related to medication administration and poor weight gain. Delamanid and bedaquiline were re-started after 10 days and were well tolerated. To complete the continuation phase, linezolid replaced clofazimine, which was felt to be the most likely cause of vomiting as enteropathy with prolonged exposure to clofazamine is well described [8] (Figure 1). At 11 months of age, low-flow oxygen was no longer required, full oral feeds were established, and a diagnosis of right hemiplegic cerebral palsy was established. Anti-tuberculosis treatment was completed at 13 months of age and the child remains well at follow-up 6 months following treatment completion.

## DISCUSSION

Diagnosis of congenital TB is difficult and relies on a high index of suspicion, especially in infants with a progressive illness unresponsive to conventional empiric therapies, born to a mother with any potential TB epidemiological exposure [7]. Bacteriological confirmation of TB is uncommon in infants and a clinical diagnosis of congenital TB may need to be made on the basis of genitourinary or disseminated TB in the mother [7]. Detection of *M. tuberculosis* from multiple sites in our case may be explained by the relative immune deficiency that affects extremely premature newborn babies, somewhat analogous to the higher sensitivity of urinary lipoarabinomannan tests for TB in severely immunosuppressed patients.

In this case, sequential use of the Xpert MTB/RIF-Ultra and MTB/XDR assays led to early escalation of anti-tuberculous therapy targeting pre-XDR TB. Xpert MB/XDR is the first automated molecular assay that detects mutations conferring resistance to drugs other than rifampin with high accuracy equivalent to line-probe assays [9]. Still, these powerful molecular platforms do not detect all resistance, as illustrated in our case by phenotypic resistance to ethionamide without a recognized mutation detected by either Xpert MDR/XDR or WGS [9].

The MDR-TB treatment landscape has evolved rapidly, with the introduction of new or repurposed drugs that enable shorter all-oral regimens [4]. To our knowledge, this is the youngest patient to receive delamanid, and this is the first publication describing the use of bedaquiline in this age group. Previous reported cases of congenital DR TB were treated with a combination of drugs including para-aminosalicylic acid, ethambutol, cyloserine, levofloxacin, kanamycin or amikacin, clofazimine, and linezolid [7]. Until recently, WHO did not recommend delamanid and bedaquiline for children <3 and 6 years of age, respectively [10]. Emerging pharmacokinetic and safety data [6] informed a new WHO recommendation published in March 2022 that bedaquiline and delamanid could be used to in children of all ages [5, 10]. We sought expert advice to extrapolate from limited paediatric data and determine doses for our patient who weighed <3 kg when these drugs were started.

Access to newer anti-tuberculous agents can be challenging, especially outside of established DR TB programs in endemic settings. There were delays in accessing oral medications infrequently used in our setting (delamanid, bedaquiline, and clofazimine). A lack of child-friendly formulations adds to the difficulty of treating DR TB in children, although new pediatric formulations of clofazimine and delamanid should become available soon [10]. We were unable to access dispersible clofazimine in a reasonable time frame, so that clofazimine (and prothionamide) suspensions were compounded in-house and replenished weekly due to uncertain stability.

Anti-tuberculous TDM has been used to explore drug pharmacokinetics (PK), pharmacodynamic (PD) targets, safety, and adherence, although rarely in the setting of congenital TB. We were able to prospectively measure antibiotic concentrations. TDM resulted in dose adjustments for amikacin, delamanid and linezolid (Supplementary Appendix). For the other drugs, TDM results reassured the treating team regarding absortion, a critical point for an unwell premature neonate with uncertain pharmacokinetic dynamics who was being treated with drugs wholly unfamiliar at this life stage. In the absence of clear PK-PD targets for most drugs we interpreted plasma concentrations against published PK studies for these agents (Supplementary Appendix).

Practical issues informed the timing and frequency of TDM. As samples for TDM had to be sent interstate to a specialised laboratory service (Pathology Queensland, Brisbane, Australia) there was a potential 7-day turnaround time for results. This laboratory performs a weekly batch run of TB TDM assays although could do more frequent testing if requested. A decision was made to perform regular TDM over the course of prolonged therapy rather than wait for treatment failure or potential drug toxicity to prompt testing as results may not have been available in a clinically useful timeframe. In addition, we could not assume steady state had been achieved with potential unpredictable changes in hepatic metabolism and excretion due to organ development over the course of treatment [11]. Drugs assays had already been developed and validated (see supplemental material for methods) and could be performed on small volume “scavenged” plasma samples.

Antenatal detection with effective treatment of TB in the mother may have prevented congenital TB and extremely premature delivery in this newborn. Despite the well-established risks of pregnancy-related TB, maternal TB is commonly not detected antenatally and often presents in the early post-partum period, as in this case [12, 13]. Although pregnancy is associated with an increased risk of developing disease in women infected with *M. tuberculosis*, clinical features are often masked by pregnancy [12, 13]. Furthermore, antenatal screening is not done especially in non-endemic settings for TB. Stigma is a barrier to establishing a history of TB treatment earlier in life [14]. Prevention and early detection of pregnancy-related TB is an important and growing area of research.

Congenital tuberculosis remains a conundrum, with a solution that must begin with improved diagnosis and management of maternal TB prior to or during pregnancy. Although diagnosis and management of affected infants is still difficult, new tools are gradually beginning to change the nature of the challenge.

## Supplementary Data

Supplementary materials are available at *Clinical Infectious Diseases* online. Consisting of data provided by the authors to benefit the reader, the posted materials are not copyedited and are the sole responsibility of the authors, so questions or comments should be addressed to the corresponding author.

## Supplementary Material

ciad540_Supplementary_Data

## Notes

***Acknowledgments.*** The authors thank the neonatal intensive care team at Joan Kirner Women's and Children's Hospital for their excellent care of this patient. They thank Lucy Sahakyan and colleagues at Otsuka Novel Products GmbH (Munich, Germany) for their support expediting the compassionate use application and delivery of delamanid. They thank Professor Simon Schaaf for his wise and rapid responses to our challenging anti-tuberculous drug dosing questions. They thank the staff of the Mycobacterium Reference Laboratory (VIDRL), Microbiological Diagnostic Unit Public Health Laboratory, The University of Melbourne, and the Victorian Tuberculosis Program. They thank the staff at Pathology Queensland, for their assistance with timely access and interpretation of therapeutic drug monitoring.
