# Supplementary material for: Pre-extensively Drug-Resistant Congenital Tuberculosis in an Extremely Premature Baby
Source: Clin Infect Dis. 2023 Sep 8;78(1):149–53. doi: 10.1093/cid/ciad540 (PMC10810709; doi:10.1093/cid/ciad540)
Supplement: ciad540_Supplementary_Data [file ciad540_supplementary_data.docx]

Contents

[Table S1. Tuberculosis Diagnostics of Mother and Baby 2](#_Toc138527866)

[Table S2. Genome Sequence Analysis 3](#_Toc138527867)

[Table S3. Therapeutic Drug Monitoring 4](#_Toc138527868)

# Table S1. Tuberculosis Diagnostics of Mother and Baby

|  | | Respiratory sampling^ | Nasogastric aspirates | Cerebrospinal fluid | | Endometrium | Blood | Urine |
| --- | --- | --- | --- | --- | --- | --- | --- | --- |
|  |  |  |  | EVD | LP |  |  |  |
| Mother | Culture | Negative | ND | Positive | Negative | Positive | Positive | Positive |
|  | PCR | Negative | ND | Positive | Negative | Positive | Positive | Positive |
| Baby | Culture | Positive | Positive | ND | Negative | ND | Positive | Negative |
|  | PCR | Positive | Positive | ND | Negative | ND | Positive | Positive |

PCR: polymerase chain reaction; ND: not done; EVD: external ventricular drain; LP: lumbar puncture ^Respiratory sampling: Mother – bronchial washings; baby – endotracheal aspirates

# Table S2. Genome Sequence Analysis

**Identification (WGS):** *M. tuberculosis*

**Phylogenetic lineage**: East-Asian (lineage 2)

**Antimicrobial Susceptibility by sequencing:**

| **Antimicrobial** | **Protein (Mutation)** |
| --- | --- |
| Rifampicin | RpoB (p.Ser450Leu) |
| Isoniazid | KatG (p.Ser315Thr) |
| Pyrazinamide | No mutation detected |
| Ethambutol | EmbB (p.Met306Val) |
| Moxifloxacin | GyrA (p.Asp94Gly) |
| Amikacin | No mutation detected |
| Cycloserine | No mutation detected |
| Ethionamide | No mutation detected |
| Para-aminosalicylic acid | ThyX (c.-16C>T),ThyA (Chromosome:g.3073680_3074470del) |

# Table S3. Therapeutic Drug Monitoring

| **Medication** | **CGA** | **Wt**  **(kg)** | **Dose** | **Duration of therapy at time of level** | **Timing of level*** | **Level (mg/L)** | **Dose adjustment** | **Comments** |
| --- | --- | --- | --- | --- | --- | --- | --- | --- |
| **Amikacin** | 30.2 | 1.1 | 20 mg/kg IV daily | Pre-6^th^ dose | Trough  (15 min pre-dose) | 4.4 | Nil | Target trough level <5 mg/L.  Target peak level 40-50 mg/L. |
|  |  |  |  | Post-6^th^ dose | Peak  (45 min post-dose) | 43.2 |  |  |
|  | 31.1 | 1.2 | ~18 mg/kg IV daily | Pre-12^th^ dose | Trough  (1 hour pre-dose) | 2.9 | Re-adjusted dose to 20 mg/kg for increased weight. |  |
|  |  |  |  | Post-12^th^ dose | Peak  (1.5 hours post-dose) | 39.1 |  |  |
| **Bedaquiline** | 45.2 | 4.1 | 10 mg PO M/W/F | 5.5 weeks (2 weeks on  30 mg daily + 3.5 weeks  on 10 mg M/W/F) | Trough  (30 min pre-dose) | 0.31 | Nil | Dose increased to 20 mg M/W/F when weight reached 5 kg.  No available target concentrations. ^a^ |
|  | 52.3 | 5.4 | 20 mg PO M/W/F | 12.5 weeks (2 weeks after increasing dose to 20 mg M/W/F) | Random  (16 hours post-dose) | 0.45 | Nil |  |
|  | 56.3 | 6.2 | 20 mg PO M/W/F | 16.5 weeks (6 weeks after increasing dose to 20 mg M/W/F) | Trough  (3.5 hours pre-dose) | 0.2 | Nil |  |
| **Clofazimine** | 40.1 | 3.1 | 5 mg/kg PO daily | 2.5 weeks | Trough  (35 min pre-dose) | 0.28 | Nil based on levels.  Dose was adjusted for increasing weight initially weekly, then every 4 weeks. | No available target concentrations. ^a^ |
|  | 42.2 | 3.4 |  | 4.5 weeks | Trough  (1.5 hours pre-dose) | 0.59 |  |  |
|  | 44.2 | 3.8 |  | 6.5 weeks | Trough  (45 min pre-dose) | 0.8 |  |  |
|  | 45.2 | 3.9 |  | 7.5 weeks | Trough  (30 min pre-dose) | 0.62 |  |  |
|  | 56.3 | 6.2 |  | 19 weeks | Trough  (3.5 hours pre-dose) | 1.16 |  |  |
| **Delamanid** | 46.6 | 4.4 | 25 mg PO daily | 15 weeks  (8 weeks on 12.5 mg daily + 7 weeks on  25 mg daily) | Trough (1 hour 20 min pre-dose) | 0.13 | AUC_0-24_ estimated to be ~4.4 mg.h/L.  Dose increased to 25 mg PO BD. | Mean AUC_0-24_ in adults reported as 7.9 mg.h/L. ^b^  AUC_0-24_ range in children reported as 2.7-12 mg.h/L. ^c^ |
|  |  |  |  |  | 2 hours post-dose | 0.36 |  |  |
|  |  |  |  |  | 4 hours post-dose | 0.26 |  |  |
|  |  |  |  |  | 8 hours post-dose | 0.17 |  |  |
| **Linezolid** | 31.0 to  39.2 | 1.1  to  2.9 | 7.5 mg/kg/dose IV or PO BD | 1.5 to 9.5 weeks | Trough (within 15 minutes to 2 hours pre-dose) | Range 0.26 to 1.85 | Dose increased to 10 mg/kg/dose BD. | Target trough level 2-5 mg/L.  Levels taken at 1-3 weekly intervals. |
|  | 40.1 to 48.2 | 3.0  to  4.6 | 10 mg/kg/dose PO BD | 10.5 to 18.5 weeks (including period on lower dose) | Trough (within 30 minutes to 1.5 hours pre-dose) | Range 1.77 to 3.31 | Dose was adjusted for increasing weight weekly. |  |
| **Moxifloxacin** | 30.4 | 1.0 | 10 mg/kg/dose IV or PO daily | 2 weeks | Trough  (2 hours pre-dose) | 0.6 | Low-level resistance detected initially at 0.25 mg/L.  Dose increased pending high-level resistance result at 1 mg/L.  Dose was adjusted for increasing weight weekly. | No available target concentrations. ^a^ |
|  | 31.0 | 1.1 | 15 mg/kg/dose IV or PO daily | 2.5 weeks | Trough  (2 hours pre-dose) | 0.3 |  |  |
|  | 33.2 | 1.5 |  | 5 weeks | Trough  (15 min pre-dose) | 0.7 |  |  |
|  | 34.3 | 1.9 |  | 6 weeks | Trough  (2 hours pre-dose) | 0.6 |  |  |
|  | 35.2 | 2.1 | 20 mg/kg/dose PO daily | 7 weeks | Trough  (3.5 hours pre-dose) | 0.6 |  |  |
|  | 36.2 | 2.3 |  | 8 weeks | Trough  (1 hour pre-dose) | 0.4 |  |  |
|  | 37.2 | 2.5 |  | 9 weeks | Trough  (15 min pre-dose) | 0.83 |  |  |

AUC_0-24_ area under plasma concentration-time curve over the last 24 hour dosing interval, BD twice daily, CGA corrected gestational age, IV intravenous, MIC minimum inhibitory concentration, M/W/F = Monday/Wednesday/Friday, PO oral (including enteral administration via orogastric or nasogastric tube), Wt weight

* The timing of blood sampling was aligned with normal clinical care to minimise the number of sampling occasions.

**Methods:**

Drug levels were measured using an Acquity ultra performance liquid chromatography system, an Acquity UPLC HSS T3 column (1.8 µM, 2.1 x 100 mm), MassLynx V4.2 software and a Xevo TQD mass spectrometer (Waters, Milford, USA). Plasma (50 µL) was mixed with 200 µL of methanol, containing deuterated internal standards for each respective drug and centrifuged for 10 minutes. The supernatant (1 µL) was injected onto the system and chromatographically resolved using a Waters 2.1 x 50 mm HSS 1.8 µm column. The mobile phases consisted of 2 mM ammonium acetate, 0.1% formic acid in water (mobile phase A) and 2 mM ammonium acetate, 0.1% formic acid in methanol (mobile phase B) and were pumped at 0.4ml/min. The column temperature was set to ambient. The gradient was as follows, after 2 minutes at initial conditions (85%A: 15%B), a linear gradient was applied to 10%A: 90%B, followed by 0%A: 100%B at 2.50 minutes and returning to initial conditions from 2.51 to 4 minutes.

Ionisation was achieved with positive mode electron spray and the mass transitions (m/z) were monitored in multiple-reaction-monitoring acquisition mode. The transitions of the parent/daughter ions were; Linezolid 338.40/ 296.30, Linezolid-d3 341.40/ 297.30, Clofazimine 473.40/ 431.20, Clofazimine-d7 480.10/ 429.20, Bedaquiline 555.30/ 555.30, Bedaquiline-d6 561.20/ 561.20, Rifampicin 823.60/ 791.60 and Rifampicin-d3 826.50/ 794.50. The dwell time was 0.01 seconds, cone voltage ranged from 25 to 45 V and the collision energy ranged from 5.0 to 30.0 eV. The capillary voltage was 0.90 kV, source temperature was 150 °C, desolvation gas low was 800 L/Hr and the collision cell gas flow (argon) at 0.40 mL/min.

Each drug was linear over the analytical range up to 50 mg/L. The coefficient of variation (%CV) ranged from 1.1% to 6.5% and 1.5% to 7.4% for intra- and inter-run respectively across 3 concentrations. Recovery for specific analytes ranged between 97% and 103%. No interferences were found when extracting plasma samples containing various other drugs encountered within our laboratory. All drugs were stable post extraction for a minimum of 24 hours,

**References:**

Alsultan A. Determining therapeutic trough ranges for linezolid. Saudi Pharm J. 2019;27(8):1061-1063.

Garcia-Prats AJ, Frias M, van der Laan L, De Leon A, Gler MT, Schaaf HS, et al. Delamanid added to an optimized background regimen in children with multidrug-resistant tuberculosis: Results of a Phase I/II Clinical Trial. Antimicrob Agents Chemother. 2022;66(5):e0214421.

Mudde SE, Upton AM, Lenaerts A, Bax HI, De Steenwinkel JEM. Delamanid or pretomanid? A Solomonic judgement! J Antimicrob Chemother. 2022; 77: 880–902.

Queensland Health: Guideline for the use of amikacin for drug resistant tuberculosis and nontuberculous mycobacterial infections in Queensland. Version 1.0 March 2021. Available at: <https://www.health.qld.gov.au/__data/assets/pdf_file/0023/1044743/tb-guideline-amikacin-drug-resistant.pdf>. Date accessed: 25/01/2023.

Sturkenboom MGG, Märtson A, Svensson EM, Sloan DJ, Dooley KE, van den Elsen SHJ, et al. Population pharmacokinetics and Bayesian dose adjustment to advance TDM of anti-TB drugs. Clinical Pharmacokinetics. 2021;60:685-710.

World Health Organization. Technical report on critical concentrations for drug susceptibility testing of medicines used in the treatment of drug-resistant tuberculosis. World Health Organization; 2018 (WHO/CDS/TB/2018.5)
